# A Retrospective Single-Center Study of Sevelamer Hydrochloride for the Treatment of Hyperphosphatemia in Children With Tumor Lysis Syndrome

**DOI:** 10.7759/cureus.33533

**Published:** 2023-01-09

**Authors:** Sawsan M Al Blewi, Aeshah A AlAzmi, Nagla Elimam, Wasil Jastaniah, Abdullah Mohammedkhalil, Shaker Abdullah

**Affiliations:** 1 Department of Pediatrics, University of Tabuk, Tabuk, SAU; 2 Department of Pediatric Oncology Hematology Bone Marrow Transplan, King Abdulaziz Medical City Jeddah, Princess Noorah Oncology Center, Jeddah, SAU; 3 Department of Pediatric Oncology Hematology Bone Marrow Transplant, King Abdulaziz Medical City Jeddah, Princess Noorah Oncology Center, Jeddah, SAU; 4 Department of Pharmacy Practice, King Abdulaziz Medical City Jeddah, Jeddah, SAU; 5 Department of Pediatric Oncology Hematology Bone Marrow Transplant, King Faisal Specialist Hospital and Research Centre, Jeddah, SAU; 6 Department of Surgery, King Khalid National Guard Hospital, Jeddah, SAU

**Keywords:** renal failure, sevelamer, hyperphosphatemia, tumor lysis syndrome, hematological malignancy

## Abstract

Introduction

Tumor lysis syndrome (TLS) is a life-threatening metabolic abnormality. The incidence of TLS depends on the underlying malignancy. In a recent analysis of hematological malignancy, the incidence of clinical TLS in children was 3.8%, laboratory TLS 46.2%, and hyperphosphatemia 32.7%. Sevelamer is effective for the treatment of hyperphosphatemia associated with renal failure; however, there is no clear data that it has the same effect in treating hyperphosphatemia with TLS.

Methods

This was a retrospective study among children aged ≤14 years with hematological malignancy who developed TLS and received sevelamer to treat hyperphosphatemia at Princess Norah Oncology Center, King Abdulaziz Medical City (KAMC) in Jeddah from January 2012 to December 2016.

Results

A total of 34 patients received sevelamer. The majority was male (64%), with a median age of six years. The median sevelamer dose per day was 1600 mg, while the median duration of use was two days. Phosphate level was significantly decreased at different times (24 hours, 48 hours, and 72 hours) during sevelamer usage, p-value <0.001.

Conclusion

In our study, the use of sevelamer resulted in a significant decrease in phosphate levels. This finding further consolidates the efficacy of sevelamer in treating hyperphosphatemia with TLS. However, further research into the drug's kinetics is recommended.

## Introduction

Tumor lysis syndrome (TLS) is a life-threatening metabolic abnormality due to the release of tumor cell components into the blood. It can happen spontaneously or due to chemotherapy administration [1]. TLS is classified as laboratory (LTLS) or clinical TLS (CTLS). LTLS recognized by two or more of the following: hyperkalemia, hyperuricemia, and hyperphosphatemia. CTLS includes LTLS plus one or more of the following symptoms: renal insufficiency, cardiac arrhythmia, and seizures [2]. The recognition of patients at hazard for having TLS is the most crucial step of management, as prophylactic measures may be initiated before TLS occur [3]. Risk factors for TLS include patients with decreased urinary flow, pre-existing hyperuricemia, renal failure, high tumor cell proliferation rate, tumor burden, and chemosensitivity [4]. The incidence of TLS differed greatly depending on the underlying malignancy. Hematologic malignancies with rapidly growing and chemosensitive cells, for example, high-grade acute lymphoblastic leukemia (ALL), carry the greatest risk [5]. More than one in four patients with that malignancy will develop TLS after treatment. Usami et al. noticed that the incidence of LTLS in hematological malignancy is 46.2%, and among these, 57.7% had hyperuricemia, 11.5% had hyperkalemia, 32.7% had hyperphosphatemia, and 34.6% had hypocalcemia [6]. Hyperphosphataemia is caused by a rapid release of phosphorous from malignant cells, which contain four times the amount of phosphorous compared to normal cells. In response, the kidney increases the urinary excretion of phosphate and decreases phosphorus reabsorption until finally the tubular transport mechanism becomes saturated and serum phosphorous levels rise. Hyperphosphataemia with other metabolic derangements can worsen the pre-existing or lead to the development of acute renal failure due to the precipitation of calcium phosphate in renal tubules [7]. Prolonged hyperphosphatemia can result in the deposition of calcium phosphate products in connective tissue, which may actively increase the danger of metastatic calcification. Management of hyperphosphatemia includes phosphorus dietary restriction using phosphorus-sequestering metallic agents (aluminum hydroxide and calcium acetate). These agents combine with dietary phosphate and convert it to an insoluble phosphate product. The main limiting adverse effect of aluminum and/or calcium-containing agents like phosphate binder is developing neurological complications, increasing calcium load, and worsening or developing renal toxicity. Using a metal-free agent as a phosphate binder is an attractive goal to increase safety while being able to effectively manage hyperphosphatemia. Sevelamer is a metal-free, non-calcium, non-aluminum-containing phosphate adhesive, which is indicated mainly for the treatment of hyperphosphatemia in chronic kidney disease patients undergoing dialysis. It binds with phosphate in the digestive tract, which decreases the amount of phosphate absorbed into the body. Its use is approved in children with end-stage renal disease to reduce serum phosphate [7]. Despite the paucity of data, some reports indicated that sevelamer might be effective for the management of hyperphosphatemia resulting from tumor lysis syndrome. Shaker and colleagues have performed a retrospective study in children with hematological malignancy and found that sevelamer is able to lower serum phosphate concentration over time significantly [8]. A couple of case studies have reported the effectiveness of the use of sevelamer in decreasing the serum phosphate level in children with/ without TLS, and they concluded that sevelamer is effective and prevents the need for hemodialysis [9-11].

Our main objective is to study sevelamer's efficacy in lowering phosphate levels secondary to TLS in children with malignancies treated in a single tertiary center.

## Materials and methods

This is a retrospective, single-center study of children ≤14 years at the Princess Noorah Oncology Center at King Abdulaziz Medical City, who had hematological malignancy with hyperphosphatemia at the time of diagnosis or after starting chemotherapy were treated with sevelamer conducted over five years (between January 2012 and December 2016). This study was approved in 2017 by the King Abdullah International Medical Research Center's (KAIMRC) protocol number # RJ17/076/J.

All patients who met the inclusion criteria have been included in the study. Patient demographic and clinical data were obtained from the medical record and Computerized Physician Order Entry (CPOE) system, which includes age, gender, diagnosis, serum white blood cell (WBC), lactate dehydrogenase (LDH), and TLS (phosphate, calcium, potassium, uric acid), calcium phosphate product and creatinine.

The following measures were collected after sevelamer administration: phosphate level at 0, 24, 48, and 72 hours; length of treatment; sevelamer dose; concurrent allopurinol and rasburicase; and need for dialysis. Hyperphosphatemia was defined as serum phosphate level ≥2.1 mmol/L or a 25% increase from baseline. Calcium phosphate product was considered a risk factor for tumoral calcinosis and calculated by multiplying the serum concentration of calcium with phosphate using the general consensus that it should not exceed 5.6 mmol/L.

We used the Cairo-Bishop criteria [4] to define the laboratory TLS: two or more laboratory changes within three days before or seven days after cytotoxic therapy, which includes a 25% increase from the baseline of uric acid, phosphate, potassium, and calcium, while the definition of clinical TLS includes laboratory evidence of laboratory TLS plus one or more of serum creatinine> 1.5 x upper limit of normal (ULN), cardiac arrhythmia / sudden death, or seizure. All patients were treated for TLS as per protocol as follows: hydration, allopurinol or rasburicase, diuretics, or dialysis if needed.

Data analysis

Descriptive analyses were used to summarize the demographic data (mean, median), while the Friedman test was run to make sure differences in pre- and post-levels of phosphate were significant before pair-wise comparisons were carried out with Bonferroni correction for multiple comparisons. Daily phosphate serum concentrations were determined and examined for three days after sevelamer administration using a linear mixed model. Analyses were conducted using the SPSS software (IBM Inc. Armonk, New York).

## Results

Thirty-four pediatric patients out of 364 who were diagnosed during the period of study with hematological malignancy received sevelamer either as a capsule or prepared from the pharmacy as a solution for patients who cannot swallow. One subject was excluded from the analysis as he expired before levels were tested at the time points. Out of the 33 patients, 66% were male and 34% were female, with a male-to-female ratio of around 3:1; the median age was six years, with a range of 2-13 years. Most of the patients (91%) were diagnosed with acute leukemia: 39% ALL B-cell, 3% relapsed b-ALL, 36% ALL T-cell, 6% acute myeloid leukemia (AML), 3% juvenile myelomonocytic leukemia (JMML), 3% Philadelphia positive ALL, and 6% lymphoma Burkitt, 3% mature T Cell lymphoma. Tumor risk stratification was 28 patients with high risks, 12 patients with intermediate risks, and four patients with low risks. The median sevelamer dose per day was 1600 mg (800 mg-3200 mg), with the median duration of use at 3.5 days (range 1-10 days). The majority of patients (73%) received rasburicase for a median duration of three days, and there was no other oral phosphate binder used during this study. Table 1 provides detailed information about patients who developed hyperphosphatemia and received sevelamer.

**Table 1:** Comparing phosphate, calcium, and calcium phosphate levels pre- and post- sevelamer treatment (n=33) ^mmol/L; ǁ using related-samples Friedman's two-way analysis of variance by ranks with 95% confidence interval

Variable	Statistics	p-value
Median phosphate level^^^ (range)		>0.0001^ǁ^
at sevelamer usage	2.34 (1.55-4.10)	
24h after sevelamer usage	1.80 (1.00-5.60)	
48h after sevelamer usage	1.43 (0.68-3.58)	
72h after sevelamer usage	1.29 (0.62-3.40)	
Median calcium level^^^ (range)		0.034^ǁ^
at sevelamer usage	2.1 (1.40-2.68)	
24h after sevelamer usage	1.94 (1.10-2.56)	
48h after sevelamer usage	2.04 (1.16-2.61)	
72h after sevelamer usage	2.1 (1.00-2.51)	
Median calcium phosphate (CaPO4) Level^^^ (range)		>0.0001^ǁ^
at sevelamer isage	4.93 (2.92-8.45)	
24h after sevelamer isage	3.40 (1.91-5.52)	
48h after sevelamer isage	2.71 (1.76-5.24)	
72h after sevelamer usage	2.60 (1.14-6.81)	

Phosphate level was significantly decreased at different time points during sevelamer usage, Ӽ2(3) = 54.487, p-value<0.001. Post hoc analysis showed a statistically significant difference from phosphate level at sevelamer usage to 24 hours (p-value=0.030), and to 48 hours (p-value< 0.0001), and to 72 hours after sevelamer usage (p-value<0.0001). There was also a statistically significant difference from 24 to 72 hours (p-value<0.001) and from 24 to 48 hours after sevelamer usage (p-value=0.040). The difference, however, was not significant from 48 to 72 hours after sevelamer usage (p-value=1.0). The difference in calcium levels was not significant at different time points during sevelamer usage, Ӽ2(3)=7.666, p-value=0.053; nevertheless, Table 2 displays phosphate, calcium, and calcium phosphate levels pre- and post-sevelamer usage.

**Table 2:** Descriptive statistics of the sample who developed tumor lysis syndrome and were treated with sevelamer (n=33) ^mmol/L; *standard deviation; Dx - diagnosis; ALL - acute lymphoblastic leukemia; AML - acute myeloid leukemia; JMML - juvenile myelomonocytic leukemia; LDH - lactate dehydrogenase; WBCs - white blood cells

Variable	Statistic
Median age in years (range)	6 (2-13)
Male sex (%)	21(64)
Diagnosis (%)	
Burkitt's lymphoma	2 (6)
Anaplastic large cell lymphoma	1(3)
T cell (ALL)	12 (36)
B cell (ALL)	13 (39)
Relapsed B cell (ALL)	1 (3)
Philadelphia (ALL)	1 (3)
M2 (AML)	1 (3)
M5 (AML)	1 (3)
JMML	1 (3)
Median serum creatinine level, μmol/L (range)	
at diagnosis	51 (30-181)
at sevelamer usage	51.50 (33-163)
Median white blood cells (WBCs), count (range)	
at diagnosis	63 (1.10-764)
at sevelamer usage	27 (1-763)
Median uric acid level^^ ^(range)	
at diagnosis	414 (75-1222)
at sevelamer usage	248.50 (60-1347)
Median LDH level (range)	
at diagnosis	2234 (114-19944)
at sevelamer usage	2136 (146-19944)
Baseline laboratory results	
Mean phosphate level^^^ at diagnosis (SD)	1.58 (±0.56)
Mean calcium (Ca) Level^^^ at diagnosis (SD)	2.29 (±0.23)
Mean potassium (K) level^^ ^at diagnosis (SD)	3.80 (±0.74)
Median urea level^ at diagnosis (range)	3.5 (1.3-16.8)
Allopurinol usage (%)	28 (85)
Median sevelamer dose per day (range)	1600 (800-3200)
Rasbricase usage (%)	24 (73)
Median rasbricase usage in days (range)	3 (0-10)

## Discussion

Hyperphosphatemia and other metabolic abnormalities secondary to TLS can lead to the development of acute renal failure due to the deposition of calcium phosphate products in connective tissue. The classical management of hyperphosphatemia includes aggressive hyperhydration and phosphorus dietary restriction using phosphorus-sequestering metallic agents (aluminum hydroxide and calcium acetate). The use of these agents is limited by the risk of neurological and renal complications [12].

Sevelamer is a metal-free, non-calcium, non-aluminum-containing phosphate adhesive used mainly for the treatment of hyperphosphatemia in chronic kidney disease patients undergoing dialysis [13]. Despite the paucity of data, a few reports with a limited number of patients indicated that sevelamer might be effective for the management of hyperphosphatemia resulting from tumor lysis syndrome. Shaker et al. [8] performed a retrospective study on 13 patients. In addition, a case report conducted by Harsha showed the effectiveness of sevelamer in treating hyperphosphatemia in two children with T-cell acute lymphocytic leukemia [9]. Another retrospective study by Kahlon et al. found that sevelamer was effective in treating hyperphosphatemia caused by tumor lysis syndrome in 17 patients [10]. To our knowledge, this report represents the largest cohort of patients to date. In the present study, we evaluated the efficacy of sevelamer in reducing the phosphate level in 33 pediatric patients with hematological malignancies who developed hypophosphatemia secondary to TLS. In addition, we estimated the incidence of renal dialysis among patients who received sevelamer, which was 10%. It has been reported that almost 20% of patients at high risk of TLS require dialysis [14]. Despite this result, the few number of patients included, the retrospective nature of the analysis, and the lack of a control group are major limitations of this study. Other important limitations include the difficulty of estimating the real contribution of sevelamer to the reduction in phosphate level, the incidence of renal failure as the standard supportive care applied to all patients at risk of TLS, and the impossibility of quantifying the role of each measure separately. We did not assess the side effects secondary to sevelamer use, although no patient was required to stop it before reducing the phosphate level. Besides those limitations, the use of sevelamer in TLS did not follow a specific protocol in our center, and a different dose range and frequency were used.

Despite all the aforesaid limitations, our study highlighted the productiveness of sevelamer in reducing the level of phosphate associated with TLS.

## Conclusions

Our study suggested that sevelamer is effective in reducing the phosphate level significantly at three time points. It also revealed that 10% of the high-risk TLS patients in our study who used sevelamer required dialysis. The results call for a large-scope study to confirm the finding and identify criteria for incorporating sevelamer as one of the prophylactic and therapeutic measures in TLS.
